# Understanding Orthorexia Nervosa: A Systematic Review of Meta-analytical Findings

**DOI:** 10.1007/s13668-025-00714-4

**Published:** 2025-12-16

**Authors:** Lorenzo Moccia, Maria Benedetta Anesini, Tommaso Callovini, Delfina Janiri, Caterina Policola, Marco Cintoni, Francesca Focà, Georgios Demetrios Kotzalidis, Fabio Conti, Adolfo Bandettini Di Poggio, Giovanni Camardese, Gabriele Sani

**Affiliations:** 1 Department of Neuroscience, Section of Psychiatry, Università Cattolica del Sacro Cuore, Largo Francesco Vito 1, 00168 Rome, Italy; 2 Department of Psychiatry, Fondazione Policlinico Universitario Agostino Gemelli IRCCS, Largo Agostino Gemelli 8, 00168 Rome, Italy; 3 Unit of Endocrinology, Department of Translational Medicine and Surgery, Università Cattolica del Sacro Cuore-Fondazione Policlinico “A. Gemelli” IRCCS, Largo Gemelli 8, 00168 Rome, Italy; 4 Department of Translational Medicine and Surgery, Catholic University of the Sacred Heart, 00168 Rome, Italy; 5 Clinical Nutrition Unit, Department of Medical and Surgical Sciences, Universitary Policlinic Agostino Gemelli Foundation IRCCS, 00168 Rome, Italy; 6 Unit of Clinical Psychology, Fondazione Policlinico Universitario Agostino Gemelli IRCCS, Università Cattolica del Sacro Cuore, 00168 Rome, Italy; 7 Istituto di Neuroscienze, Neomesia Kos Group, Via Nomentana 1362, 00137 Roma, Italy; 8 Department of Life Science, Health, and Health Professions, Link Campus University, Via del Casale di S. Pio V, 44, 00165 Rome, Italy

**Keywords:** Orthorexia nervosa, Eating disorder spectrum, Perfectionism, Compulsive exercise, Obsessive-compulsive tendencies

## Abstract

**Abstract:**

Orthorexia Nervosa (ON) is an emerging condition marked by an obsessive focus on healthy eating, often resulting in impairments in well-being, daily functioning, and quality of life. Despite growing awareness, ON is not currently recognized in major psychiatric diagnostic systems, complicating its identification and clinical management.

**Objective:**

This umbrella review aims to synthesize evidence from existing meta-analyses on the prevalence and associated factors of ON, while critically assessing the quality of the current literature.

**Methods:**

A comprehensive review of five meta-analyses was conducted, focusing on ON prevalence rates, its psychological correlates, and the quality of the included studies. The review also evaluated heterogeneity in findings and the reliability of assessment tools used across studies.

**Results:**

Prevalence estimates of ON showed wide variability due to differences in populations and assessment instruments. ON was significantly associated with compulsive exercise, eating disorder symptoms, obsessive-compulsive traits, and perfectionism. Newer diagnostic tools reveal a stronger connection between ON and obsessive-compulsive tendencies, while distinguishing it from traditional eating disorders, due to its focus on food purity rather than weight control. Methodological limitations, such as inconsistent assessment tools and variable study designs, limit the conclusiveness of current evidence, rendering most findings suggestive rather than definitive.

**Discussion:**

The review highlights the need for standardized diagnostic criteria, validated screening instruments, and longitudinal studies to establish the clinical relevance of ON. Enhancing understanding of its psychological and behavioral foundations is crucial for improving diagnostic accuracy and developing effective interventions.

**Supplementary Information:**

The online version contains supplementary material available at 10.1007/s13668-025-00714-4.

## Introduction

Orthorexia nervosa (ON) is an emerging condition within the realm of disordered eating, characterized by an extreme preoccupation with healthy eating that significantly impacts an individual’s well-being and quality of life [1]. In the review by Pontillo et al. [2], all included studies found that individuals with ON report lower levels of well-being, reduced life satisfaction, higher stress, more pronounced depressive symptoms, and lower overall functioning compared to individuals without ON. Despite its increasing recognition, ON is not currently classified as an official diagnosis in major psychiatric manuals such as the Diagnostic and Statistical Manual of Mental Disorders, Fifth Edition (DSM-5) or the International Classification of Diseases, 10th Revision (ICD-10) [3–5]. This diagnostic ambiguity complicates the identification and management of ON, contributing to its under-recognition in clinical settings.

The phenomenon of ON is often contrasted with “healthy orthorexia,” a non-pathological interest in maintaining a healthy diet that does not result in distress or functional impairment [6]. In contrast, ON involves a rigid adherence to dietary rules and an obsessive focus on the perceived “purity” of food, which can lead to medical complications such as malnutrition and severe emotional distress [7]. Subjects engaging in orthorexic behavior use mainly eating patterns that can be summarized as the following (Table 1): “High-sugar products & snacks”, “Fatty products & Dressings (e.g., salad dressings)”, “Oils & potatoes”, (avoided) and “Meat” (selectively avoided or chosen) and “Fresh products & nuts”, and “Dairy products & whole-meal bread” (chosen) [8]. These patterns reflect “healthy” and “unhealthy” eating practices and preoccupation with food quality [9, 10].

**Table 1:** Patterns of eating practices followed by people with orthorexia nervosa

Avoided	Chosen
Chocolate and candies	Fresh vegetables and fruit (especially low-sugar)
Biscuits	Nuts
Salty snacks, ultra-processed (junk food)	White meat
Sweetened foods and drinks	Whole grain bread
Cheese	Seeds
Red meat	Olive oil
Creams, butter and margarine	Dairy products
Animal fats	
Potatoes	
Fried food	
Oils (except olive oil)	

Given the substantial variability in reported rates and the potential for severe psychological and physical outcomes, a comprehensive understanding of ON is essential. Although several meta-analyses have examined different aspects of ON, including its prevalence, psychological correlates, and social implications, to date, no umbrella review has synthesized these findings. Therefore, the aim of this article is to provide a systematic overview and critical evaluation of meta-analytic research on ON. This umbrella review seeks to clarify the rates and correlates of ON, assess the quality of existing evidence, and identify gaps in the current literature. By doing so, we hope to offer a clearer perspective on the clinical relevance of ON and highlight directions for future research and diagnostic refinement.

## Materials and Methods

### Eligibility Criteria

We included meta-analyses investigating ON. To be eligible, studies had to: report prevalence rates or correlates related to ON using validated measures; focus on meta-analyses that provided quantitative estimates, such as effect sizes, odds ratios, or correlation coefficients; include clear definitions of ON and its assessment tools. In cases of overlapping data across meta-analyses examining the same outcomes, we prioritized the one with the largest number of studies included, the most comprehensive findings, or the most recent publication date.

### Search Strategy

A systematic literature search for meta-analyses was conducted on the PubMed, Scopus, and Google Scholar databases on July 31, 2025. No language restrictions were applied. Gray literature, conference abstracts, and all publications not having undergone peer-review were excluded. Our search strategy included search in title of “orthorexia OR orthorectic OR orthorexic” and meta-analysis as an article type. After a preliminary screening based on titles and abstracts, full texts were retrieved to evaluate eligibility. Articles were independently screened and read in full text by three authors and any potential disagreement was resolved by discussion with a fourth author.

### Data Extraction

Two authors independently extracted data and blindly cross-checked them for accuracy. A data extraction template was used to collect key information from the eligible studies, including: author(s) and year of publication; number of included trials (K); total sample size (N); main details needed to assess the risk of bias; measures of the effects with their 95% confidence intervals (95%CIs), p-values, and heterogeneity measures (I^2^ statistic values).

### Risk of Bias Assessment

The Risk of Bias in Systematic Review (ROBIS) tool was used to assess the risk of bias of the included systematic reviews. There are three phases in ROBIS, including assessing relevance, identifying concerns with the review process, and judging risk of bias [11]. Phase one of ROBIS tool includes one item, which mainly evaluates whether the participants, exposures, comparators and outcomes match the target question. The answers are “yes,” “no,” “partial,” and “uncertain”. Phase two includes four domains: (1) study eligibility criteria; (2) identification and selection of studies; (3) data collection and study appraisal; (4) synthesis and findings [11]. The signaling questions in phase two are answered as “yes,” “probably yes,” “probably no,” “no” and “no information”. Based on the answer to each signaling question, the bias associated with each domain is then judged as “low,” “high,” or “unclear.” Phase three considers whether the systematic review as a whole is at risk of bias [11]. In this phase, the following questions were considered: (1) did the interpretation of findings addressed all the concerns identified in domains 1 to 4; (2) was the relevance of identified studies appropriately considered in review’s research question; (3) did the reviewers avoid emphasizing the results on the basis of their statistical significance? [11].

The answers to these signaling questions are the same as phase two. Based on the answers to the questions in phase three, the overall risk of bias in the systematic reviews were rated as “low,” “high,” or “unclear.” Two investigators independently evaluated the risk of bias of all the included systematic reviews, and the disagreements were resolved by discussion.

### Quality of Evidence

The classification system used in this study was based on the criteria outlined by Fusar-Poli and Radua [12] with the primary goal of assessing the robustness of evidence across multiple meta-analyses. The system categorizes evidence into four classes (Class I - Convincing, Class II - Highly Suggestive, Class III - Suggestive, and Class IV - Weak) based on statistical parameters such as sample size, *p*-values, heterogeneity (I²), prediction intervals, and potential biases. Additionally, a fifth category, “not significant,” was applied to studies that did not demonstrate statistical significance (*p* > 0.05). The classification criteria for each class are as follows:

- Class I (Convincing): Requires more than 1,000 cases, *p*-value less than 10⁻⁶, I² below 50%, a 95% prediction interval excluding the null value, and the absence of both small-study effects and excess significance bias.
- Class II (Highly Suggestive): Requires more than 1,000 cases, a *p*-value less than 10⁻⁶, and statistically significant results from the largest included study, while not meeting the stricter criteria for Class I.
- Class III (Suggestive): Requires more than 1,000 cases and a *p*-value less than 10⁻³, without fulfilling the criteria for Classes I and II.
- Class IV (Weak): Requires a *p*-value less than 0.05 and not meeting the criteria for Classes I to III.
- Not significant: Applies to studies where the *p*-value exceeds 0.05.

The classification process involved a detailed review of the key parameters reported in each meta-analysis. These included the total number of participants across studies, reported *p*-values, heterogeneity (I²) levels, and 95% prediction intervals. Additionally, analyses of small-study effects were conducted using funnel plots and Egger’s tests to evaluate publication bias. The presence of excess significance bias was assessed where possible. Each included meta-analysis was evaluated according to these criteria. For studies that did not report prediction intervals explicitly, this factor was noted as a limitation in their classification. Moreover, the robustness of the evidence was further analyzed based on the contribution of the largest study within each meta-analysis. Studies with high heterogeneity (I² >50%) were considered less robust and could not qualify for Class I or Class II, even if other criteria were met. The final classifications were determined by synthesizing all reported parameters and applying the classification system uniformly across all included studies. The goal was to ensure consistency in evaluating the strength and reliability of the evidence presented in each meta-analysis.

## Results

Our search on July 31, 2025, produced 62 records. At the end of the eligibility process, we included five studies. All included studies were written in English, although this was not a prerequisite. The full results of our search are shown as a PRISMA flowchart in the supplementary material (Figure S1)**.** Description of the included studies, including information on study population, study design, assessment tools for ON and index outcome are reported in Table 2.

**Table 2:** Characteristics of included studies

Author(s), year	Study population	Study design	Assessment of ON	Assessment of index outcomes
Strahler et al., 2021 [16]	The study populations included competitive, elite, and recreational athletes, general populations (convenience samples), and individuals with a specific interest in fitness, such as gym attendees and sports science students. The study included a total of **10**,**134 participants (56.4% female)**, reflecting a diverse demographic profile. Participants came from various countries and cultural backgrounds, with no regional restrictions. While no strict age limits were applied, most participants were young adults (mean age = 25.21 years). Populations of both genders with gender breakdowns are included. Participants with exclusive diagnoses of other eating disorders like anorexia nervosa or bulimia nervosa were excluded	Systematic review and meta-analysis	Studies employed a version of the ORTHO test for the diagnosis of ON; other studies used the Düsseldorf Orthorexia Scale (DOS) or the Eating Habits Questionnaire (EHQ). Specifically, 68% of the studies used the ORTO-15 or its variations, while fewer studies employed the DOS (3 studies) or the EHQ (2 studies).	Tests to assess exercise addiction (ExAdd): • **Exercise Dependence Scale (EDS)** • **Exercise Addiction Inventory (EAI)** • **Scale of Dedication to Exercise (SDE)** • **Compulsive Exercise Test (CET)**: **Tests to assess physical activity**: • **International Physical Activity Questionnaire (IPAQ)** • **General Practice Physical Activity Questionnaire (GPPAQ)** • **Metrics based on weekly training hours**,** exercise intensity**,** and participation in organized sports**
Zagaria et al., 2021 [17]	The study population comprised a total of 16,097 participants across all studies. Recruitment strategies predominantly involved convenience sampling or targeting specific groups, such as students, community samples, or individuals identified as being at risk for ON. Most participants had a BMI within the healthy weight range (18.5–24.9 kg/m²). Women constituted the majority of the sample (70.3%). Participants were primarily adults aged 18 years or older, with a mean age ranging from 19.41 to 67.9 years. Eligibility criteria included a diagnosis of ED or OCD, while no other psychiatric conditions were required	Systematic review and meta-analysis; however, many of the studies included in the meta-analysis are cross-sectional observational studies	· **ORTO-15**: The most commonly used instrument (25 studies), sometimes in modified versions. · **Düsseldorf Orthorexia Scale (DOS)**: Used in 5 studies. · **Eating Habits Questionnaire (EHQ)**: Used in 3 studies. · **Bratman Orthorexia Test (BOT)**: Used in 2 studies. · **Orthorexia Nervosa Inventory (ONI)**: Used in 1 study	To assess e**ating disorders (EDs)**: • **Eating Attitudes Test (EAT-40 and EAT-26)**: Used 22 studies. • **Eating Disorder Examination Questionnaire (EDE-Q)**: Used in 9 studies. • **Eating Disorder Diagnostic Scale (EDDS)** and **Eating Disorders Inventory-3 (EDI-3)**: Used in 1 study each **To assess Obsessive-Compulsive Symptoms (OCD)**: • **Obsessive-Compulsive Inventory-Revised (OCI-R)**: Used in 11 studies. • **Maudsley Obsessive-Compulsive Inventory (MOCI)**: Used in 4 studies. • **Yale-Brown Obsessive-Compulsive Scale (Y-BOCS)**: Used in 2 studies.
Huynh et al., 2023 [18]	The study population consisted of a total of 17,813 participants across 40 studies. The populations investigated encompassed university students, dietitians, fitness enthusiasts, vegans/vegetarians, healthcare professionals, and clinical samples diagnosed with disorders such as obsessive-compulsive disorder (OCD) or eating disorders (ED). A majority of the studies reported a predominantly female cohort, with 27 out of 40 studies including over 65% female participants. The mean age of the participants was 24.87 years.	Systematic review and meta-analysis	· **ORTO-15 and its variants** · **Eating Habits Questionnaire (EHQ)** · **Bratman Orthorexia Test (BOT)** · **Düsseldorf Orthorexia Scale (DOS)** · **Teruel Orthorexia Scale (TOS)** · **Orthorexia Nervosa Inventory (ONI)** · **Test of Orthorexia Nervosa (TON)** · **Orthorexia Nervosa Scale (ONS)**	To assess Obsessive-Compulsive Symptoms (OCD): • **Obsessive-Compulsive Inventory-Revised (OCI-R)** • **OCI-12** • **Yale-Brown Obsessive-Compulsive Scale (Y-BOCS)** • **Obsessive-Compulsive Symptoms Checklist**
Lopez-Gil et al., 2023 [14]	The study population consisted of a total of 30,476 participants from 75 studies conducted across different regions, including Europe (35 studies), Asia (18), South America (12), North America (5), and Oceania (1). The participants represented diverse groups, including the general population, individuals focused on sports performance or body composition, people from health-related programs or professions, individuals living with specific diseases, and those following special diets. Women constituted the majority of the sample (61.2%), and participants ranged in age from 13 to 93 years. Studies exclusively focused on individuals with diagnosed eating disorders such as anorexia nervosa, bulimia nervosa or other psychiatric disorders were excluded.	Systematic review and meta-analysis	**ORTO-15** with a cutoff of < 35 or < 40 points. · **34 studies** used the **< 35 cutoff** exclusively. · **64 studies** used the **< 40 cutoff** exclusively. · **23 studies** reported results using **both cutoff values (< 35 and < 40 points).**	To examine the role of sex, type of population, mean age, BMI, and temporal trends in relation to ON symptoms, the study employed a combination of subgroup analyses and meta-regression techniques.
Pratt et al., 2024 [19]	The study population comprised a total of 7,064 participants across all studies. The majority of the studies were conducted in an educational setting, primarily involving undergraduate students, with some including postgraduate students or vegan/vegetarian participants. Other studies were conducted in sports/exercise domains, and one in a general community context. The population was predominantly female (5,815 participants), with only four studies including mostly male participants (1,072 participants). Most samples had a mean age in their twenties (4,874 participants), some studies focused on participants in their thirties (1,262 participants), one study reported a mean age of nineteen (459 participants), and another study included participants aged 35–54 years (469 participants).	Systematic review and meta-analysis	· **ORTO-15 and Variants** · **Eating Habits Questionnaire (EHQ)** · **Teruel Orthorexia Scale (TOS)** · **Düsseldorf Orthorexia Scale (DOS)**: · **Revised-Bratman Orthorexia Test (rBOT)** · **Orthorexia Nervosa Inventory (ONI)**	To assess perfectionism, the following scales were utilized: · **Frost Multidimensional Perfectionism Scale (F-MPS)** · **Hewitt and Flett Multidimensional Perfectionism Scale (HF-MPS)** · **Almost Perfect Scale (APS)** · **Big Three Perfectionism Scale (BTPS)** · **Sport Multidimensional Perfectionism Scale 2 (S-MPS-2)**

### Prevalence of ON Symptoms

Three meta-analyses were found which reported the rates of ON [13–15]. According to our methodology, the study by López-Gil et al. [14] was selected. The summary of findings on the prevalence of ON are reported in Table 3. López-Gil et al. [14] reported an overall prevalence of ON of 27.5% (95% CI = 23.5–31.6, I^2^ = 97%, K = 34) among 19,652 individuals. The subgroup analysis in relation to sex reported that the proportion of ON symptoms for female sex was 34.6% (95% CI = 29.5–39.8, I^2^ = 96.1%, K = 18), and for male sex, it was 32.1% (95% CI = 26.5–38.1, I^2^ = 93.1%, K = 16), with no significant differences between sexes (*P* = 0.550). A subgroup analysis regarding the type of population (i.e., general population, people focused on sports performance or body composition, people from health-related programs or professions, people living with the disease, or people with a special diet) was performed. The highest overall proportion was found in people focused on sports performance or body composition (34.5%, 95% CI = 23.1–47.0, I^2^ = 98.0%, K = 8) without significant differences in comparison with other types of populations (*P* = 0.692). Moreover, a subgroup analysis according to the data collection year of the studies (i.e., prior to 2016, between 2016 and 2019, or between 2020 and 2023) found no statistically significant differences were found (*P* = 0.293), but rather a temporal increase in the proportions of ON symptoms over the years, with a tendency towards a higher prevalence in the most recent studies (i.e., 2020 to 2023) (31.7%, 95% CI = 25.4 to 38.3, I^2^ = 94.9%, K = 8).

**Table 3:** Prevalence of orthorexia nervosa symptoms among different groups

Author(s), year	Index group	K	*N*	Prevalence (%)	95%CI	*P*-value	Heterogeneity (I² %)
López-Gil et al., 2023 [14]	Overall	34	19,652	27.45	23.48–31.61	Na	97.0
	Female	18	8,004	34.65	29.47–39.83	0.550	96.1
	Male	16	5,308	32.14	26.5–38.1		93.1
	General population	14	9,526	24.62	18.62–31.14	0.692	97.94
	People from health-related programs or professions	6	3,376	25.77	20.54–31.36		91.87
	People focused on sports performance or body composition	8	3,104	34.53	23.05–46.99		97.97
	People living with the disease	3	2,605	26.53	24.10–29.03		0
	People with a special diet	3	1,041	26.27	16.66–37.18		91.81
	Study with data collection before 2016	15	9,761	24.47	18.49–30.98	0.293	97.53
	Study with data collection between 2016 and 2019	11	4,772	28.68	21.32–36.66		96.62
	Study with data collection between 2020 and 2023	8	5,119	31.65	25.37–38.28		94.91

95%CI = 95% Confidence Interval; k = number of included studies; N = number of included subjects; *Na* Not Applicable

### ON Correlates

**Fig. 1:**
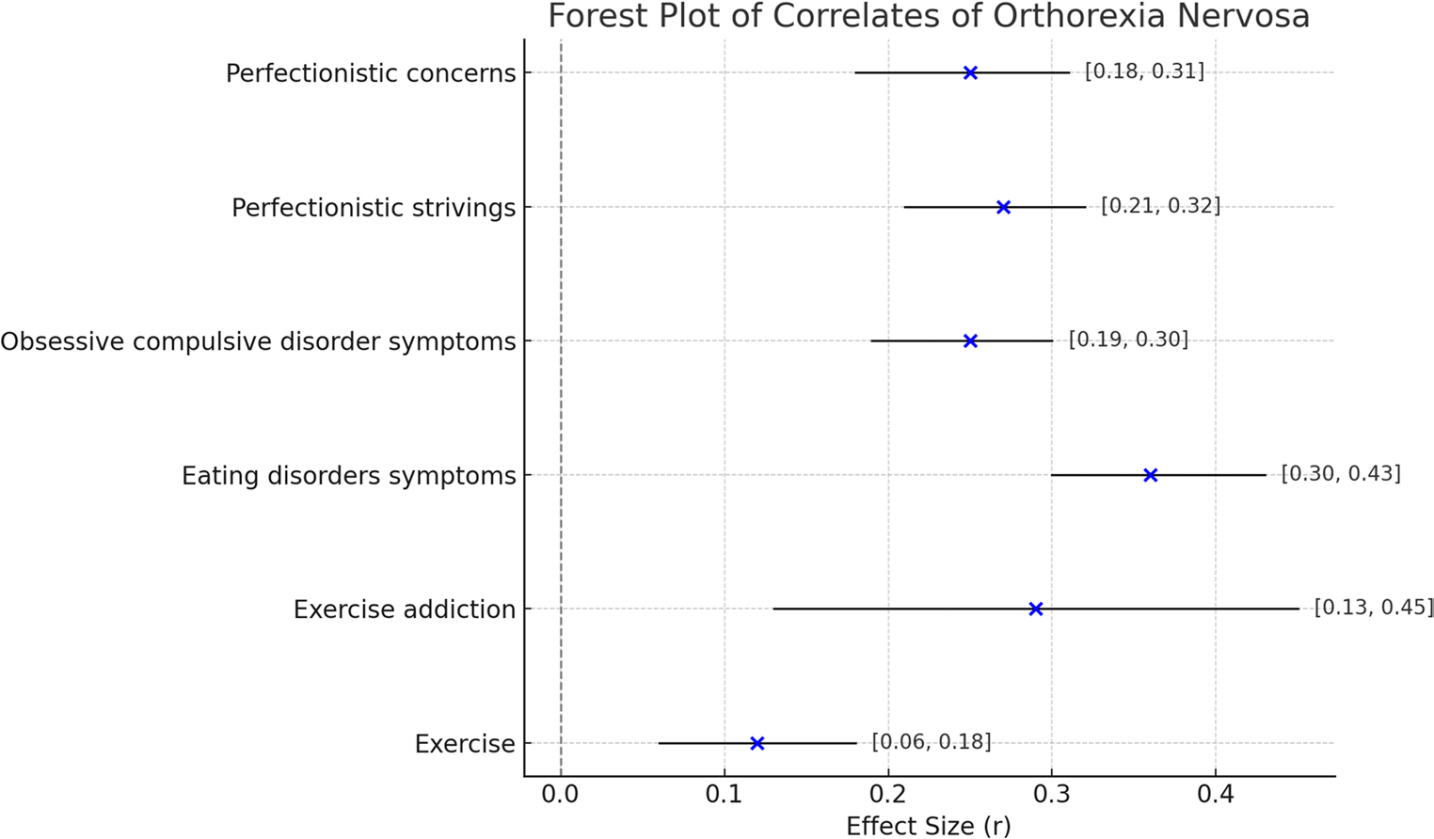
Forest plot summarizing the effect sizes of key correlates of orthorexia nervosa

The summary of findings on correlates of ON symptoms are reported in Table 4 and Fig. 1.

**Table 4:** Correlates of orthorexia nervosa

Author(s), year	Correlates of orthorexia nervosa	K	*N*	Effect Size (*r*)	95%CI	*P*-value	Heterogeneity (I² %)
Strahler et al., 2021 [16]	Exercise	21	9,292	0.12	0.06–0.18	< 0.001	84.21
	Exercise addiction	7	2,791	0.29	0.13–0.45	< 0.001	90.41
Zagaria et al., 2021 [17]	Eating disorders symptoms	33	14,549	0.36	0.30–0.43	< 0.0001	95.96
Huynh et al., 2023 [18]	Obsessive compulsive disorder symptoms	31	12,356	0.25	0.19–0.30	< 0.001	89.00
Pratt et al., 2024 [19]	Perfectionistic strivings	11	3,956	0.27	0.21–0.32	< 0.05	61.06
	Perfectionistic concerns	13	4,984	0.25	0.18–0.31	< 0.05	74.99

95%CI = 95% Confidence Interval; k = number of included studies; N = number of included subjects

#### Association Between Exercise or Exercise Addiction and ON

One meta-analysis was identified that examined the association between exercise or exercise addiction and ON [16]. In the context of exercise, the average correlation across 21 studies (*n* = 9,292) demonstrated a small effect size (Pearson’s *r* = 0.12) with considerable heterogeneity (I² =84.21%). When stratified by ON measurement instruments, studies utilizing the DOS or EHQ scales reported a slightly higher correlation (Pearson’s *r* = 0.19) compared to those employing the ORTO scale (Pearson’s *r* = 0.09). However, the difference between these groups was not statistically significant (*p* = 0.116). Furthermore, the association between exercise and ON was comparable between men and women, with no significant difference detected (*p* = 0.088). In the context of exercise addiction or compulsive exercise, the average correlation across seven studies (*n* = 2,791) revealed a medium effect size (Pearson’s *r* = 0.29), again with high heterogeneity (I² = 90.41). Due to the limited number of studies, subgroup analyses based on ON measurement instruments and gender could not be performed.

#### Association Between Eating Disorder Symptoms and ON

A meta-analysis of 33 studies (*n* = 14,549) examined the association between ON and eating disorder symptoms [17]. The random-effects model revealed a moderate overall effect size (Pearson’s *r* = 0.36, 95% CI [0.30–0.43], *p* < 0.0001), with high heterogeneity (I² = 95.96%). Sensitivity analyses excluding gray literature (including dissertations) did not substantially alter the findings (Pearson’s *r* = 0.37, 95% CI [0.30–0.43], *p* < 0.0001, I² = 96.14%). Subgroup analysis by study quality also yielded consistent results: studies with a quality score ≥ 5 reported an effect size of Pearson’s *r* = 0.35 (95% CI [0.27–0.43]), while those with a quality score < 5 showed a slightly higher effect size of Pearson’s *r* = 0.41 (95% CI [0.29–0.53]). Further subgroup analyses by ON instrument type found moderate associations for studies using the ORTO scale (Pearson’s *r* = 0.34), the EHQ (Pearson’s *r* = 0.39), and the DOS (Pearson’s *r* = 0.25). High heterogeneity persisted across all subgroups. Analyses by weight status (BMI 18.5–24.9 kg/m²) showed a pooled effect size of Pearson’s *r* = 0.33 (95% CI [0.26–0.40]), and cultural context analysis revealed stronger associations in Western countries (Pearson’s *r* = 0.43) compared to Eastern countries (Pearson’s *r* = 0.23). No significant publication bias was detected in any analyses.

#### Association Between Obsessive Compulsive Disorder Symptoms and ON

Two meta-analyses were identified that examined the association between obsessive-compulsive (OC) symptoms and ON [17, 18]. In line with our methodology, we selected Huynh et al. [18]. Their random-effects meta-analysis revealed a significant overall correlation of Pearson’s *r* = 0.25 (95% CI [0.19–0.30], *p* < 0.001). High heterogeneity was observed across the studies (I² = 88.6%). Subgroup analyses compared assessments developed before and after the updated diagnostic criteria proposed by Dunn & Bratman [3]. Specifically, assessments developed after 2016 showed a stronger correlation (Pearson’s *r* = 0.40, 95% CI [0.33–0.46], *p* < 0.001, I² = 84%) compared to those developed before 2016 (Pearson’s *r* = 0.22, 95% CI [0.16–0.28], *p* < 0.001, I² = 89%). This difference was statistically significant (*p* < 0.001) and not attributable to variations in internal consistency between the two groups of assessments.

#### Association Between Perfectionism and ON

A meta-analysis of 18 studies, conducted by Pratt et al. [19], included 19 samples (*n* = 7,064) and investigated the relationship between multidimensional perfectionism—perfectionistic strivings (PS) and perfectionistic concerns (PC)—and ON, highlighting their distinct, yet interconnected, influences on eating behaviors. This meta-analysis revealed that PS and PC exhibited a medium-to-large positive relationship with each other (*r*⁺ = 0.48, 95% CI [0.25–0.65]). Both PS and PC showed small-to-medium positive relationships with orthorexia (PS: *r*⁺ = 0.27, 95% CI [0.21–0.32]; PC: *r*⁺ = 0.25, 95% CI [0.18–0.31]). After controlling for the relationship between perfectionism dimensions, PS (*r*⁺ = 0.20, 95% CI [0.11–0.26]) remained a significant predictor of orthorexia, whereas PC did not (*r*⁺ = 0.12, 95% CI [− 0.03–0.26]). Substantial heterogeneity was detected in the weighted mean effect sizes, prompting moderation analyses based on age, gender, domain, orthorexia instrument, and perfectionism instrument. About PS meta-regression showed that neither age (β = 0.15, *p* = 0.66) nor gender (β = −0.56, *p* = 0.05) moderated the relationship between PS and orthorexia. Subgroup analyses revealed no significant differences across domains, orthorexia instruments, or perfectionism instruments. Regarding PC, meta-regression indicated that age (β = −0.03, *p* = 0.92) and gender (β = −0.05, *p* = 0.87) did not moderate the relationship between PC and orthorexia. Subgroup analyses showed no differences across domains or perfectionism instruments. However, mixed evidence suggested differences based on orthorexia instruments.

### Risk of Bias

The ROBIS tool was used to assess the risk of bias of the included systematic reviews. According to the results of phase 1, in all the included studies participants, exposures, comparators, and outcomes matched the target question. The results of phase 2 and 3 are shown in the supplementary material (Figure S2).

### Quality of Evidence

The quality assessment of the included studies revealed that most of them fall within Class III (Suggestive) according to the established classification system. This is primarily due to the high heterogeneity (I² >50%) and the inclusion of the null value within the 95% prediction intervals, indicating inconsistent findings across potential new studies. Specifically, the studies by López-Gil et al. [14], Strahler et al. [16] and Zagaria et al. [17] were classified as Class III due to their large sample sizes (> 1,000 cases) and statistically significant results (*p* < 10⁻³), but they failed to meet the stricter criteria for Classes I and II due to elevated heterogeneity and other methodological limitations. Conversely, the studies by Pratt et al. [19] and Huynh et al. [18] achieved Class II (Highly Suggestive) classification. These studies reported robust sample sizes, highly significant *p*-values (*p* < 10⁻⁶), and statistically significant results in the largest included studies. However, they did not meet the requirements for Class I (Convincing) due to high heterogeneity (I² >50%) and the lack of explicit confirmation that the 95% prediction intervals excluded the null value. Overall, while the included studies provide meaningful insights, the presence of high heterogeneity and methodological limitations restricts their generalizability, indicating that further research with more consistent methodologies is necessary to strengthen the evidence base. The full classification process is reported in the supplementary material (Table S1).

## Discussion

The findings from this umbrella review highlight insights regarding the prevalence and correlates of ON as well as methodological limitations revealed through our quality assessment and risk of bias analysis.

### Prevalence of ON

The prevalence of ON symptoms varied widely across the included meta-analyses. López-Gil et al. [14] reported an overall prevalence of 27.5%, with substantial heterogeneity (I² = 97%). This variability in prevalence estimates can be attributed to differences in population characteristics, assessment tools, and temporal factors. Our quality assessment revealed that these prevalence estimates were primarily based on studies classified as Class III (Suggestive) evidence, indicating that the findings, while statistically significant, are not robust due to high heterogeneity and methodological concerns. This underscores the need for standardized diagnostic criteria to ensure more reliable prevalence estimates. The ROBIS analysis showed that most studies had a low overall risk of bias. However, the review by López-Gil et al. [14] was rated as unclear due to insufficient reporting on the robustness of findings. The main issues identified included inconsistent application of eligibility criteria and limited consideration of between-study heterogeneity. These biases can impact the accuracy of prevalence estimates and suggest that future research should prioritize clearer methodological reporting. López-Gil’s et al. study [14], which included 30,476 individuals from 18 countries, further explored ON prevalence patterns. Results indicated that 27.5% of participants exhibited ON symptoms according to the ORTO-15 questionnaire, with higher prevalence among athletes, fitness enthusiasts, and individuals highly concerned with body composition. No significant gender differences were observed (32.1% in men and 34.6% in women), contrasting with previous studies that reported a higher incidence in women. The highest prevalence was found among those particularly focused on sports performance or body composition, reaching 34.5%. López-Gil et al. [14] also raise the possibility of an inverse causal relationship, suggesting that rather than sports participation increasing orthorexic symptoms, individuals with pre-existing orthorexic tendencies may be more likely to engage in sports and body composition control as a means of performance enhancement. Moreover, the study indicates a progressive increase in ON incidence over time, as evidenced by recent research conducted between 2020 and 2023 [20]. This rising trend could be attributed to the widespread use of social media and its influence in promoting specific dietary regimens and often unrealistic body ideals. Additionally, new social and cultural habits have emerged, emphasizing the pursuit of an ideal psychophysical health status closely linked to dietary control and body image regulation. Although prevalence estimates vary significantly due to differences in study populations and assessment methodologies, findings consistently suggest that ON symptoms are widely present across diverse demographic groups.

###  Correlates of ON

The association between exercise and ON was examined by Strahler et al. [16], who reported a small but significant correlation (*r* = 0.12) with high heterogeneity (I² = 84.21%). The strength of the association varied depending on the measurement tool used, with the DOS and EHQ scales showing a slightly higher correlation compared to the ORTO scale. This finding aligns with the hypothesis that individuals focused on physical performance may be more prone to developing ON behaviors. Our quality assessment rated this study as Class III (Suggestive) evidence, reflecting the presence of high heterogeneity and small effect sizes. Nevertheless, the ROBIS analysis indicated a low risk of bias, suggesting that despite these limitations, the findings are relatively reliable and consistent with the existing literature. Strahler et al. [16] hypothesized the existence of a correlation between ON and the practice of physical exercise, as both behaviors are often the result of a heightened focus on health and well-being. Specifically, they propose that this correlation is stronger in cases where physical activity is compulsively or progressively increasing, compared to those who maintain a balanced training routine. The underlying idea is that the relationship between ON and compulsive exercise may lead to a behavioral pattern characterized by rigid dietary control and an obsession with body composition, where exercise serves as a means of weight and body shape regulation. According to the authors, this correlation may be more pronounced in women, as excessive physical exercise is frequently associated with eating disorders, which predominantly affect the female population. In men, however, physical activity might be driven by different factors, less directly linked to dietary control, as observed in muscle dysmorphia, also known as reverse anorexia. This disorder is characterized by a pathological obsession with increasing muscle mass and achieving a defined physique, leading individuals to perceive themselves as excessively thin or underdeveloped, despite already possessing significant muscularity [21]. The habitual exposure to sports environments tends to reinforce the importance of a strict diet and the pursuit of the “ideal” physique, which may foster obsessive behaviors related to food control and training frequency, thereby increasing the risk of ON. Finally, Strahler et al. [16] explore whether compulsive exercise should be included in the diagnostic criteria for ON, but ultimately conclude that it should be considered a risk factor rather than an essential characteristic of the disorder. This distinction is crucial because, although their meta-analysis confirms a connection between these two phenomena, it does not support a complete overlap between ON and compulsive exercise.

Zagaria et al. [17] explored the relationship between ON and eating disorder symptoms (EDs), revealing a moderate effect size (*r* = 0.36) with high heterogeneity (I² = 95.96%). The association was consistent across different study quality levels and measurement tools, indicating that ON shares several features with traditional eating disorders. Our quality assessment classified this study as Class III (Suggestive) evidence due to the high heterogeneity observed across studies. The ROBIS tool flagged a low risk of bias, although concerns about publication bias were noted. According to the analysis conducted by Zagaria et al. [17], there appears to be a significant overlap between ON and eating disorders (ED). However, this correlation is not strong enough to justify a complete assimilation of ON into traditional ED, leading the authors to hypothesize that ON may represent an emerging and distinct condition deserving of an independent characterization. One of the primary differences between ON and ED lies in the focus of the pathological preoccupation: while ED are primarily concerned with food quantity and weight control, ON is centered on the quality and purity of food. Despite this distinction, both conditions share a high degree of behavioral rigidity and a pathological obsession with specific dietary choices, which may result in dangerous dietary restrictions. Despite these concerns, the findings provide meaningful insights into the overlap between ON and other eating disorders, highlighting the need for further research to differentiate these conditions.

The relationship between obsessive-compulsive symptoms (OCs) and ON was examined by Huynh et al. [18]. The meta-analysis found a significant correlation (Pearson’s *r* = 0.25) with high heterogeneity (I² = 88.6%). Subgroup analyses revealed that more recent assessments of ON show a stronger association with OCs, possibly due to improved diagnostic criteria. Our quality assessment classified this study as Class II (Highly Suggestive) evidence, indicating that despite high heterogeneity, the findings are robust. The ROBIS analysis rated this study as having a low overall risk of bias, further supporting the reliability of the results. According to Huynh et al. [18], ON shares several characteristics with Obsessive-Compulsive Disorder (OCD), including recurrent and intrusive thoughts about food, its quality, purity, or potential contamination. Furthermore, rigid rituals associated with food preparation and consumption resemble the compulsions typically observed in OCD. However, while OCD encompasses a broad range of obsessions and compulsions across various domains of daily life, ON is specifically centered on the concept of healthy eating and food preparation rituals that exhibit obsessive-compulsive traits [2]. Unlike OCD, where compulsive behaviors are performed to alleviate distress, in ON, the ritualized preparation of food serves to maintain dietary behaviors that align with one’s personal beliefs and values. Additionally, according to Janas-Kozik et al. [22] the obsessive fixation on food quality and preparation methods, along with a meticulous focus on details related to food composition and appearance, as well as the avoidance of certain storage or cooking methods, positions ON within the OCD spectrum. However, the debate remains open, as some researchers argue that ON constitutes a distinct condition characterized by high levels of perfectionism and an intense need for control, features that align it more closely with eating disorders than with OCD.

The strong link between OC traits and ON suggests that cognitive-behavioral interventions targeting obsessive-compulsive tendencies may be effective in managing ON. Cognitive-Behavioral Therapy (CBT) is a structured, evidence-based approach aimed at modifying dysfunctional thoughts and rigid behaviors characteristic of ON. According to Horovitz and Argyrides [23], CBT helps individuals recognize and reframe extreme beliefs related to food, nutrition, and body image while developing strategies to manage anxiety and distress associated with deviations from strict dietary patterns. A key aspect of CBT is fostering greater flexibility in food choices, allowing for the gradual reintroduction of previously avoided foods and promoting a healthier, more balanced relationship with eating. Additionally, continuous monitoring of eating habits and associated emotional responses enables the identification of triggers that reinforce orthorexic behaviors, increasing self-awareness and facilitating long-term behavioral change.

Perfectionism, particularly perfectionistic strivings (PS), was identified as a significant correlate of ON. Studies found a medium-to-large positive relationship between perfectionism and ON, with PS (Pearson’s *r* = 0.27) being a stronger predictor than perfectionistic concerns (PC) (Pearson’s *r* = 0.25). Our quality assessment classified this evidence as Class III (Suggestive) due to the high heterogeneity across studies. Despite this, the ROBIS tool rated the overall risk of bias as low, indicating that the results are generally reliable. The findings suggest that individuals with high perfectionistic tendencies may be at greater risk for ON.

Pratt et al. [19] hypothesize that in the case of ON, perfectionism manifests differently compared to other ED. Rather than being driven by anxiety about making mistakes, fear of external judgment, or the discrepancy between idealized standards and body perception, it is characterized by a rigid obsession with achieving an absolute ideal of health. This translates into extreme control over dietary habits and a relentless pursuit of a “perfect” way of eating. Unlike disorders such as anorexia and bulimia, where perfectionism is primarily focused on attaining an ideal weight and body shape, in ON, the main motivation appears to be strict adherence to self-imposed dietary rules, perceived as essential for well-being. ON therefore seems to be less associated with self-criticism and more with a form of self-directed perfectionism, in which individuals impose severe dietary restrictions on themselves, not necessarily due to a fear of social judgment. Those affected develop an extreme level of control over their diet, meticulously planning meals, eliminating entire food groups, and refusing to eat food prepared by others out of fear that it may not meet their strict health criteria. This rigidity is fueled by the belief that only a perfectly regulated diet can ensure optimal health. Unlike anorexia, where self-esteem is often tied to weight loss, in ON, a sense of personal accomplishment derives from the ability to strictly adhere to a pure and rigorous dietary regimen. Perfectionism, in this context, takes the form of a personal challenge rather than a response to social pressure or fear of judgment. As a result, many individuals with ON do not perceive their behavior as problematic but rather as a demonstration of willpower and determination. This lack of awareness distinguishes ON from other ED, where psychological distress and self-criticism are typically more pronounced. From a treatment perspective, therapeutic interventions may need to shift the focus from merely reducing performance anxiety to enhancing cognitive flexibility. Helping patients develop a more balanced relationship with food and reduce the rigidity of their beliefs could be a crucial goal. In particular, it may be beneficial to address their perception of health and well-being, emphasizing that the pursuit of perfect health is an unrealistic goal and that some degree of dietary flexibility is essential for overall physical and mental well-being. Interventions aimed at addressing maladaptive perfectionism could be beneficial in reducing ON symptoms.

Although definitive diagnostic criteria and standardized assessment tools are still lacking [24–26], recent evidence suggests that treatments developed for ED may be the most effective for ON [26]. This is because ON shares a common psychopathological core with ED, OCD, perfectionism, and compulsive exercise. This core is characterized by rigid and dichotomous thinking, an excessive need for control, and difficulties in emotional regulation, which are also central features of the other aforementioned psychopathological conditions.

From a neurobiological perspective, Cartwright [24] observed that the levels of serotonin, endorphins, norepinephrine, and cholecystokinin (CCK) are often reduced in patients with ED, leading to mood disturbances, decreased physical and emotional satisfaction, and a diminished sense of satiety after meals. Furthermore, individuals with ED exhibit elevated cortisol and vasopressin levels, hormones typically associated with chronic stress and emotional distress.

Similar to ED, ON involves a pathological relationship with food. However, while anorexia nervosa and bulimia nervosa are characterized by control over food quantity and an obsession with body weight, ON manifests as an obsession with the purity and quality of food. This preoccupation can lead to significant nutritional deficiencies, malnutrition, and weight loss due to an extremely selective and unbalanced diet [25]. Overall, Strahler & Stark [26], suggest that individuals with ON exhibit worsened physical health, with tangible risks of osteopenia, hormonal imbalances, and severe nutritional deficiencies.

Due to the overlap between multiple clinical entities, ON may be part of a continuum of disorders characterized by a false sense of control, where food intake, binge eating, or restrictive dieting function as strategies to manage emotional distress and reduce anxiety [24].

The relationship between ON and OCD is evidenced by the presence of recurring intrusive thoughts and accompanying compulsive rituals, such as rigid meal preparation and consumption patterns, which lead to marked anxiety when deviating from self-imposed dietary rules. Thomas et al. [27] demonstrated that ED and OCD share alterations in fronto-striatal and limbic circuits, which are involved in cognitive and emotional regulation. Specifically, increased activity in the orbitofrontal cortex and anterior cingulate cortex has been observed, areas responsible for habit formation, impulse regulation, and error evaluation. These mechanisms could explain the cognitive and behavioral rigidity characteristic of ON. Perfectionism, often associated with obsessive-compulsive traits, plays a key role in ON, as it fuels the pursuit of a “perfect” diet or ideal food, promoting unrealistic and unattainable standards. Additionally, compulsive exercise can take on the characteristics of a true compulsion, serving as a body control strategy to achieve an idealized state of health. This behavior has significant endocrine implications, including hyperactivity of the hypothalamic–pituitary–adrenal (HPA) axis, frequently observed in ED leading to elevated cortisol levels and the maintenance of a chronic stress state [28].

Overall, ON can be understood as a distinct psychiatric disorder, a symptom of another eating disorder, or a contemporary social phenomenon. However, it is increasingly evident that ON results from a complex interplay of neurobiological, psychological, and environmental factors, requiring further research to establish clear diagnostic criteria and develop an appropriate multidisciplinary management approach.

### Limitations

This review has some limitations that should be acknowledged. First, the high heterogeneity observed across studies limits the generalizability of the findings. Differences in study populations, assessment tools, and methodological approaches contribute to the variability in results. Second, the reliance on different diagnostic tools for ON, such as the ORTO-15, DOS, and EHQ scales, complicates comparisons across studies. These instruments vary in their validity and reliability, leading to inconsistent prevalence estimates and correlates. Another limitation is the potential presence of publication bias. While sensitivity analyses were conducted to mitigate this risk, it is possible that some findings were inflated due to selective reporting. Additionally, the lack of longitudinal data limits our understanding of the progression and long-term impact of ON. Most studies included in this review were cross-sectional, which restricts the ability to infer causality between ON and its correlates. Finally, cultural and contextual factors were not consistently addressed in the included studies. ON may manifest differently across populations, influenced by cultural norms and societal pressures. Future research should prioritize culturally sensitive approaches to better capture the nuances of ON in diverse populations.

### Recommendations for Future Research

Our findings emphasize the need for future research to develop and validate standardized diagnostic tools that are reliable and culturally adaptable, which is essential for accurately assessing ON. Addressing the issue of high heterogeneity is another critical priority, as more focused studies with consistent methodologies and clearly defined populations are necessary to reduce variability between studies. Additionally, mitigating publication bias by encouraging the publication of null findings and ensuring comprehensive reporting will significantly improve the reliability of the existing evidence base. Finally, conducting longitudinal studies will help to understand the progression of ON symptoms over time and provide valuable insights into potential risk factors and intervention points that could be addressed in clinical practice.

## Conclusions

In conclusion, our quality assessment and risk of bias analysis highlight significant methodological limitations in the existing evidence base for ON. The high heterogeneity and risk of bias observed across studies emphasize the need for more rigorous research practices. Future research should focus on reducing bias and improving the reliability of prevalence estimates and correlates, ultimately enhancing the clinical relevance of ON findings and supporting evidence-based interventions.

As ON becomes increasingly prevalent and its associations with perfectionism, compulsive exercise, and obsessive-compulsive traits become clearer, it is imperative to establish standardized diagnostic criteria and targeted treatment approaches. The ongoing debate over whether ON should be classified as a distinct disorder or as part of an existing spectrum (such as ED or OCD) remains a critical challenge. Addressing this ambiguity will be essential for improving clinical recognition, guiding therapeutic interventions, and ensuring appropriate support for affected individuals. Additionally, the growing impact of social media, diet trends, and health movements on eating behaviors underscores the importance of a multidisciplinary approach. Psychological, nutritional, and medical expertise must converge to develop strategies that promote a balanced relationship with food while preventing the rigid, harmful patterns associated with ON. Establishing clearer classification criteria and validated assessment tools will be a crucial step toward refining future research and improving clinical practice.

## Key References

- Strahler J, Wachten H, Mueller-Alcazar A. Obsessive healthy eating and orthorexic eating tendencies in sport and exercise contexts: A systematic review and meta-analysis. J Behav Addict. 2021;10(3):456–70. doi: 10.1556/2006.2021.00004. https://doi.org/10.1556/2006.2021.00004.
  - ○ An important contribution addressing physical exercise in orthorexia. Participants are from various countries, adding to the generalizability of results. The population consists of both athletes, sport science students or gym goers.
- Huynh PA, Miles S, de Boer K, Meyer D, Nedeljkovic M. A systematic review and meta-analysis of the relationship between obsessive-compulsive symptoms and symptoms of proposed orthorexia nervosa: The contribution of assessments. Eur Eat Disord Rev. 2024;32(2):257–80. https://doi.org/10.1002/erv.3041.
  - ○ This important paper explored the relationships between OCD-like symptoms and orthorexia. This meta-analysis involved a large number of studies and participants and included a wide range of participants.
- Pratt VB, Hill AP, Madigan DJ. Multidimensional perfectionism and orthorexia: a systematic review and meta-analysis. Eat Weight Disord. 2024;29(1):67. https://doi.org/10.1007/s40519-024-01695-z.
  - ○ An important contribution on the relationship between orthorexia and perfectionism. Most included studies were conducted in school environments and included vegetarian and vegan participants, as well as sportsmen.

## Supplementary Information

Below is the link to the electronic supplementary material.

Supplementary File 1 (DOCX 81.0 KB)

## Data Availability

Not applicable. All data are in the manuscript/supplemental material and in cited articles.
