# Supplementary material for: Understanding Orthorexia Nervosa: A Systematic Review of Meta-analytical Findings
Source: Curr Nutr Rep. 2025 Dec 16;14(1):126. doi: 10.1007/s13668-025-00714-4 (PMC12708686; doi:10.1007/s13668-025-00714-4)
Supplement: Supplementary file 1 — (DOCX 81.0 KB) [file 13668_2025_714_MOESM1_ESM.docx]

**SUPPLEMENTARY MATERIAL**

**Figure S1.** Search results

**Identification of studies via databases and registers**

Records identified thought database searching (PUBMED):

(n = 62)

**Identification**

**Screening**

Records excluded (n = 57):

- **Lack of relevance (n=37)**
- **Ineligible population (n=10)**
- **Insufficient data (n=5)**
- **Qualitative approach/case reports (n=5)**

Records screened

(n =62)

Records assessed for eligibility

(n = 5)

Records included (n = 5):

- **Provided relevant quantitative data**
- **Used validated tools**
- **High methodological quality**
- **Focus on the main topic**

Studies included in review

(n = 5)

**Included**

*Consider, if feasible to do so, reporting the number of records identified from each database or register searched (rather than the total number across all databases/registers).

**If automation tools were used, indicate how many records were excluded by a human and how many were excluded by automation tools.

*From:*  Page MJ, McKenzie JE, Bossuyt PM, Boutron I, Hoffmann TC, Mulrow CD, et al. The PRISMA 2020 statement: an updated guideline for reporting systematic reviews. BMJ 2021;372:n71. doi: 10.1136/bmj.n71.

### Phase 1: Identification

**Records Identified:**
A PubMed/Scopus/Google Scholar search on 31/7/2025 using the query "orthorexia OR orthorectic OR orthorexic" in the title yielded **62 records** filtered for "Reviews," "Systematic Reviews," and "Meta-Analyses."

**Duplicates Removed:**
No duplicates were identified, leaving a total of **62 records**.

### Phase 2: Screening

**Exclusion by Title and Abstract:**
**Reasons for excluding 57 articles:**

- **Lack of specificity on the topic:**
  Many articles addressed general eating disorders or excessive physical exercise without focusing directly on orthorexia nervosa (ON).
- **Qualitative approach:**
  Some articles were qualitative studies or case reports without quantitative data or meta-analyses.
- **Unvalidated tools:**
  Several studies did not use recognized tools such as ORTO-15 criteria to evaluate ON.
- **Ineligible population:**
  Studies conducted on adolescents, children, or individuals with psychiatric diagnoses unrelated to ON.

### Phase 3: Eligibility (Full-Text Evaluation)

**Records Assessed for Eligibility:**
Five full-text articles were analyzed.

**Reasons for Including the 5 Articles:**

- **Provided relevant quantitative data:**
  For example, effect sizes and statistical correlations between ON and other disorders.
- **Used validated tools:**
  Included instruments such as ORTO-15 or comparable tests to identify ON.
- **High methodological quality:**
  Studies with a low risk of bias and transparent methodology.
- **Focus on the main topic:**
  The relationship between ON and obsessive-compulsive symptoms, eating disorders, and specific behaviors.

**Full-Text Exclusions:**
No articles were excluded at this stage, as the 5 selected articles met all the inclusion criteria.

### Phase 4: Inclusion

**Studies Included in the Final Review:**
The meta-analysis included **5 articles**:

- **Strahler et al. 2021 [13]**
- **Zagaria et al., 2021 [14]**
- **Huynh et al., 2023 [15]**
- **López-Gil et al., 2023 [11]**
- **Pratt et al., 2024 [16]**

### Specific Exclusion Criteria

**57 Articles Excluded for the Following Reasons:**

- **Lack of relevance (37 articles):**
  Focused on eating disorders, exercise, or healthy diets without a specific focus on ON.
- **Ineligible population (10 articles):**
  Included studies on adolescents, children, or individuals with other psychiatric diagnoses.
- **Insufficient data (5 articles):**
  Did not provide adequate statistical information or used unrecognized tools to evaluate ON.
- **Qualitative approach or case reports (5 articles):**
  Not suitable for a systematic review or meta-analysis.

**Figure S2.** Risk of bias assessment

| **RISK OF BIAS DOMAINS** | **Phase 2** | | | | **Phase 3** |
| --- | --- | --- | --- | --- | --- |
|  | STUDY ELIGIBILITY CRITERIA | IDENTIFICATION AND SELECTION OF STUDIES | DATA COLLECTION AND STUDY APPRAISAL | SYNTHESIS AND FINDINGS |  |
| **Study ID.** |  |  |  |  |  |
| **Orthorexia Nervosa (ON)** | | | | | |
| Zagaria et al. (2021) [14] | 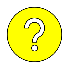 | 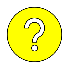 | 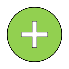 | 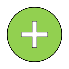 | 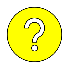 |
| Strahler et al. (2021) [13] | 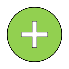 | 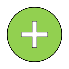 | 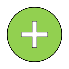 | 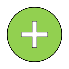 | 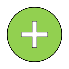 |
| López-Gil et al. (2023) [11] | 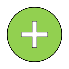 | 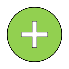 | 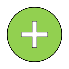 | 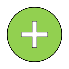 | 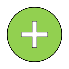 |
| Huynh et al. (2023) [15] | 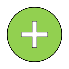 | 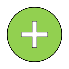 | 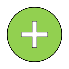 | 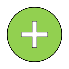 | 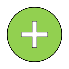 |
| Pratt et al. (2024) [16] | 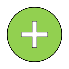 | 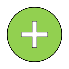 | 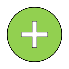 | 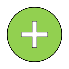 | 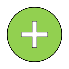 |
| Judgment: 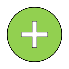Low; 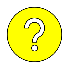 Unclear; 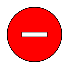 High | | | | | |

**Table S1.** Quality Assessment Classification System

| **Class I - Convincing** | **Class II – Highly Suggestive** | **Class III -Suggestive** | **Class IV -Weak** | **Non-significant** |
| --- | --- | --- | --- | --- |
| - Number of cases >1000 - *p* < 10⁻⁶ - I² < 50% - 95% prediction interval excluding the null value - absence of small-study effects - absence of excess significance bias | - Number of cases >1000 - *p* < 10⁻⁶ - the largest study shows a statistically significant effect - criteria for Class I are not met | - Number of cases >1000 - *p* < 10⁻³ - criteria for Classes I–II are not met. | - *p* < 0.05 - criteria for Classes I–III are not met | - *p* > 0.05 |

**Convincing (Class I):** when the number of cases >1000, *p* <10⁻⁶, I² < 50%, 95 % prediction interval excluding the null value, absence of small-study effects, and absence of excess significance bias.

**Highly Suggestive (Class II):** when the number of cases >1000, *p* <10⁻⁶, the largest study shows a statistically significant effect, and the criteria for Class I are not met.

**Suggestive (Class III):** when the number of cases >1000, *p* < 10⁻³, and the criteria for Classes I–II are not met.

**Weak (Class IV):** when *p* < 0.05 and the criteria for Classes I–III are not met.

**Not significant:** when *p* > 0.05.

### Strahler et al., 2021 [13]

| **Number of Cases** | **P-Value** | **Heterogeneity (I²)** | **95% Prediction Interval (PI)** | **Small-Study Effects and Excess Significance Bias** | **Class** |
| --- | --- | --- | --- | --- | --- |
| 10,134 participants in total for included studies | Reported  p-values for the associations:  **ON-general exercise (Ex):**  p < 0.001 with an effect size r = 0.12 (95% CI: 0.06-0.18).  **ON- addictive exercise (ExAdd):**  p < 0.001 with an effect size r = 0.29 (95% CI: 0.13-0.45) | I² = 84.21% for overall exercise correlation (**Ex)**  I² = 90.41% for addictive exercise correlations (**ExAdd**). | **For the correlation between ON and general exercise (Ex)**  PI: [−0.03,0.27]  **For the correlation between ON and addictive exercise (ExAdd)**  PI: [−0.23,0.81] | The analyses for small-study effects (funnel plot and Egger's test) do not indicate strong publication bias. However, the limited number of studies in some subsets (n=7 for ExAdd) restricts the robustness of this assessment. Additionally, no issues of excess significance bias are reported. | **Class III (Suggestive)** due to the large number of cases (n > 1000), significant p-values (p < 10^-3),^ but high heterogeneity (I² > 50%) and lack of null value exclusion in prediction intervals. |

### Classification Analysis

**Convincing (Class I):** Fails due to high heterogeneity (I² > 50%), p-value that does not meet the stricter threshold of p < 10⁻⁶, and the absence of explicitly reported prediction intervals that exclude the null value.

**Highly Suggestive (Class II):** Fails due to the high heterogeneity, the prediction intervals include the null value, indicating that the results are not consistent for new studies, and it is unclear whether the largest study contributes significantly or dominates the overall findings."

**Suggestive (Class III):** Number of cases >1,000 and p < 10^-3^ are met. It does not meet the stricter requirements for Classes I or II due to high heterogeneity and prediction intervals that include the null value, indicating inconsistent results across potential new studies. Additionally, it is unclear whether the largest study contributes significantly or dominates the overall findings. These factors align the article with the criteria for Class III.

**Weak (Class IV):** Not applicable, as the significance exceeds p < 0.05.

### Zagaria et al., 2021 [14]

| **Number of Cases** | **P-Value** | **Heterogeneity (I²)** | **95% Prediction Interval** | **Small-Study Effects and Excess Significance Bias** | **Class** |
| --- | --- | --- | --- | --- | --- |
| 16,097 participants in total for included studies.  **ON-EDs association**: 14,549 participants across 33 studies.  **ON-OCD association**: 7,778 participants across 17 studies. | Reported p-values for the associations:  **ON-EDs:** p < 0.0001 with an effect size r = 0.36 (95% CI: 0.30-0.43)  **ON-OCDs:** p < 0.001 with an effect size r = 0.21 (95% CI: 0.15-0.27) | **I² = 95.96%** for **ON and EDs.**  **I² =** 86.47 for **ON and OCDs**. | The prediction intervals are not explicitly mentioned but appear less robust due to high heterogeneity. | Funnel plot inspections and Egger's regression tests showed no significant publication bias, suggesting a lower likelihood of small-study effects. The largest studies confirm statistically significant effects for both associations ON-EDs and ON-OCDs. | **Class III (Suggestive)** due to number of cases >1000, p < 10⁻^3^ and all criteria for Class I and II are not met. |

### Classification Analysis

**Convincing (Class I):** Fails due to high heterogeneity (I² > 50%), p-value that does not meet the stricter threshold of p < 10⁻⁶, and the absence of explicitly reported prediction intervals that exclude the null value.

**Highly Suggestive (Class II):** Fails because the p-value does not meet the stricter threshold of p < 10⁻⁶ required for this class, despite having a large sample size and significant results. Additionally, high heterogeneity (I² > 50%) further disqualifies it.

**Suggestive (Class III):** The overall classification as Class III is based on the combined evidence from the two primary associations: ON-EDs and ON-OCD. While the ON-EDs association is stronger, the ON-OCD association qualifies due to its smaller effect size (r = 0.21) and high heterogeneity, which disqualifies it from higher classifications. Furthermore, the article demonstrates substantial heterogeneity (I² > 50%) across both associations. As the overall strength of the evidence is diluted by the weaker ON-OCD findings, the article as a whole aligns best with Class III. This classification reflects a moderate level of evidence, with statistically significant results but limited consistency and generalizability.

**Weak (Class IV):** Not applicable, as the significance level exceeds **p < 0.05**.

**Huynh et al., 2023 [15]**

| **Number of Cases** | **P-Value** | **Heterogeneity (I²)** | **95% Prediction Interval (PI)** | **Small-Study Effects and Excess Significance Bias** | **Class** |
| --- | --- | --- | --- | --- | --- |
| 17,813 participants in total for included studies | The study reports multiple significant correlations between **ON and OCs**. **p < 10⁻³** is frequently reported, and some correlations show **p < 10⁻⁶**, as evidenced by studies using valid instruments (TOS, ONI). | Heterogeneity is reported at **88.6%,** which is **much higher than the 50% threshold** for Class I.  Also, the study discusses variability across measurement tools (ORTO-15 versus more recent scales like TOS and ONI). | The prediction interval is not specifically mentioned. The absence of this data means that the criterion for Class I is not met. | A formal analysis of small-study bias is not conducted. However,82.5% of the included studies found significant relationships between ON and OC symptoms, these ranged from small to large correlations.  There is also no specific mention of excess significance bias. | **Class II (Highly Suggestive)** Due to the number of cases exceeding 1,000, p < 10⁻⁶ being met for key results, and significant effects reported in the major studies included, the study aligns with Class II criteria. |

**Classification Analysis**

**Convincing (Class I):** It does not meet the stricter criteria for Class I due to high heterogeneity, the lack of specific mention of prediction intervals, and insufficient assessment of bias.

**Highly Suggestive (Class II):** The criteria for the number of cases >1,000 and p < 10⁻⁶ are satisfied.

Significant effects are consistently reported in the largest studies, supporting this classification. The article’s evidence quality aligns with Class II given the robust number of cases and statistically significant results. However, high heterogeneity and the lack of confirmation for critical criteria preclude it from being classified as Class I.

**Suggestive (Class III):** Not applicable, as the criteria for Class II are satisfied.

**Weak (Class IV):** Not applicable, as the significance level far exceeds p < 0.05.

**López-Gil et al., 2023 [11]**

| **Number of Cases** | **P-Value** | **Heterogeneity (I²)** | **95% Prediction Interval (PI)** | **Small-Study Effects and Excess Significance Bias** | **Class** |
| --- | --- | --- | --- | --- | --- |
| 30,476  participants in total for included studies | The reported p-value is ***p* < 10⁻⁶** for the overall analysis. | High heterogeneity was reported (I² = 97.0%), which exceeds the 50% threshold required for Class I criteria. | The study does not explicitly report a prediction interval excluding the null value. | The study uses the Doi plot and LFK index to assess publication bias and small-study effects. It reports no asymmetry, indicating the absence of small-study effects. Also, it finds no evidence of excess significance bias.  There is no **explicit confirmation** that the largest study demonstrates a statistically significant effect. | **Class III (Suggestive)** due to number of cases >1000, p < 10⁻^3^, I² = 97.0%, the absence of explicit prediction intervals excluding the null, and missing detailed results from the largest study. |

### Classification Analysis

**Convincing (Class I):** The study does not qualify for Class I due to high heterogeneity (I² = 97.0%) and the lack of explicit 95% prediction intervals excluding the null. While it meets other criteria, these issues prevent it from reaching Class I.

**Highly Suggestive (Class II):** While the study meets the majority of the requirements, **the lack of explicit confirmation about the largest study showing a statistically significant effect** disqualifies it from Class II.

**Suggestive (Class III):** Class III is assigned because the study fulfills the basic thresholds of having a sufficient sample size (>1,000) and highly significant results (p < 10⁻³). However, it does not meet the more stringent criteria for Class I or Class II due to high heterogeneity (I² = 97.0%), the absence of explicit prediction intervals excluding the null, and the lack of detailed results from the largest study.

**Weak (Class IV):** Not applicable, as the significance exceeds p < 0.05.

**Pratt et al., 2024 [16]**

| **Number of Cases** | **P-Value** | **Heterogeneity (I²)** | **95% Prediction Interval (PI)** | **Small-Study Effects and Excess Significance Bias** | **Class** |
| --- | --- | --- | --- | --- | --- |
| The total number of participants across all studies is 7,064. Of these, a subset of 4,984 participants was included in the meta-analysis. | The document reports various p-values associated with different analyses.  **Perfectionistic strivings (PS) -ON**, p = 0.66 for age and p = 0.05 for gender.  **Perfectionistic concerns (PC) -ON**, p = 0.92 for age and p = 0.87 for gender.  Additionally, in the subgroup analysis on **ON measurement instruments**, p<0.001, which is the most relevant and comprehensive result for a key part of the study. | The meta-analysis reports varying levels of I², indicating differences in effect size consistency.  For **Perfectionistic strivings (PS)**, (I²=61.06%) but varied by domain, in the education domain (I²=70.08%) and in the sport/exercise domain (I²=8.04%).  **Perfectionistic concerns (PC)** (I²=74.99%), with significant variability in the sport/exercise domain (I²=93.00%) and in education (I²=53.45%).  **ON measurement instruments** also influenced I² levels significantly (QB=19.62), with instruments like TOS-ON showing no I². | The article reports that both PS and PC have positive relationships with ON.  For **PS**, the effect size is r+=0.27 with a 95% confidence interval of [0.21, 0.32], while for **PC**, the effect size is r+=0.25 with a 95% confidence interval of [0.18, 0.31]. These confidence intervals suggest significant positive associations as they do not include the null value. However, the article does not explicitly state whether the 95% **prediction intervals** exclude the null value. | Publication bias was evaluated using Egger’s regression and other methods. However, the study does not confirm or deny the presence of small-study effects explicitly. Also, it does not explicitly discuss whether excess significance bias was present. The largest included study also shows statistically significant results, fulfilling a key criterion for Class II. | **Class II (Highly Suggestive)** because it includes a large sample of participants and reports highly significant results p < 10⁻⁶  for the relationships between perfectionistic traits and ON. While robust, it does not meet Class I criteria due to heterogeneity (I² > 50%) and lack of confirmation that the 95% prediction interval excludes the null. |

### Classification Analysis

**Convincing (Class I):** Excluded due to lack of verifiable data for I², 95% prediction interval, small-study effects, and excess significance bias.

**Highly Suggestive (Class II):** Satisfied. The study has >1000 cases, p < 10⁻⁶, and statistically significant results from the largest study. All other criteria for Class II are met.

**Suggestive (Class III) and Weak (Class IV):** These are not applicable because the study exceeds the requirements for Class II.
